# The Partnering with Patients Model of Nursing Interventions: A First Step to a Practice Theory

**DOI:** 10.3390/healthcare3020252

**Published:** 2015-04-24

**Authors:** Wendy Moyle, Claire M. Rickard, Suzanne K. Chambers, Wendy Chaboyer

**Affiliations:** 1 Centre for Health Practice Innovation, Menzies Health Institute Queensland, Griffith University, Nathan Campus QLD 4111, Australia; E-Mails: c.rickard@griffith.edu.au (C.M.R.); suzanne.chambers@griffith.edu.au (S.K.C.); w.chaboyer@griffith.edu.au (W.C.); 2 NHMRC Centre of Research Excellence in Nursing (NCREN), Menzies Health Institute Queensland, Griffith University, Nathan and Gold Coast Campus, QLD 4111, Australia; 3 Preventative Health, Griffith Health Executive, Griffith University, Gold Coast Campus, QLD 4222, Australia

**Keywords:** nursing interventions, nursing theory, nursing model, complex healthcare interventions, patient centred care, capabilities approach

## Abstract

The development of a body of knowledge, gained through research and theory building, is one hallmark of a profession. This paper presents the “Partnering with Patients Model of Nursing Interventions”, providing direction towards how complex nursing interventions can be developed, tested and subsequently adopted into practice. Coalescence of understanding of patient-centred care, the capabilities approach and the concept of complex healthcare interventions led to the development of the model assumptions and concepts. Application of the model to clinical practice is described, including presentation of a case study, and areas for future research including understanding both patients’ and nurses’ perceptions and experiences when the model is in use, and testing the effect of nursing interventions based on the model are recommended.

## 1. The Partnering with Patients Model of Nursing Interventions: A First Step to a Practice Theory

A profession has been defined as “an occupation whose incumbents create and explicitly utilize systematically accumulated general knowledge in the solution of problems posed by a clientele” [1]. The development of a body of knowledge, gained through research and theory building is one hallmark of a profession [1,2,3]. This body of knowledge is influenced by the knowledge from other disciplines, sometimes termed ‘borrowed knowledge’ [4,5], yet it remains crucial that practice disciplines such as nursing develop their own unique knowledge base [5,6]. Importantly, the recent exponential growth in nursing research has contributed to nursing as a unique scientific discipline with its own language and knowledge. While in recent years, nursing research has maintained an agenda of evidence-based patient care and the research outcomes have contributed to translation into practice and policy, developing new nursing theories alongside this empirical knowledge is needed to help the profession to identify knowledge strengths and gaps and guide the future direction of clinical practice, future research and nursing education. Importantly, new nursing theory can explain what nurses do and why and in doing so potentially reduce conflict between the care team through a consistent approach; allow care to be mutually understood by patients and families as well as other healthcare professionals; improve patient care; and enhance professional status.

In nursing, the development of theories has taken place on a number of levels [4]. Meta-theory refers to the theory of theory and is focused at the “big” philosophical and methodological level. Grand theories provide a conceptual framework that emphasises broad perspectives on practice but these are abstract and difficult to test [4]. Middle-range theories are the bridge between grand theories and practice theories. Such theories present concepts and propositions at a lower level of abstraction, they only deal with specific phenomena and a limited number of variables ensuring they are narrower in scope than grand theories, but still have a reasonably broad perspective [4,7]. Finally, practice theories have a limited scope and level of abstraction as they explore one particular situation found in nursing practice [4,7], the essence of which has been described as “a desired goal and prescriptions for actions to achieve the goal” [4]. A new level, termed situation-specific theory has emerged, with the intent to more closely link theory to research [8,9,10]. Situation-specific theories focus on specific phenomena and practices, and may be limited to specific populations [8].

A conceptual model, another tool for theory building, is defined as “a set of relatively abstract and general concepts that address the phenomena of central interest to a discipline, the propositions that broadly describe those concepts and the propositions that state relatively abstract and general relations between two or more concepts [7]. This paper presents a conceptual model, including the model concepts, that is situation-specific to nursing interventions, entitled the “Partnering with Patients Model of Nursing Interventions” (PPM-NI). While not yet developed to the extent of a theory, this model provides a preliminary understanding of how complex nursing interventions can be developed, tested and subsequently adopted into practice. The use of models or theories to underpin healthcare interventions is advocated by various bodies and groups and promotes the importance of combining theory with research in order to produce nursing science that is generalizable, logical and used by nursing practitioners to guide and improve practice [11,12,13]. Trends in the delivery of care, methodological literature and the researchers’ own experience has led to the development of this model, which can be integrated into a research intervention and to advance nursing practice. Empirical observation, scholarly insight and deduction are used to develop models [7] and these factors were important in the development of the model described. The PPM-NI model is first outlined, and then its applicability to nursing research and practice is demonstrated with the use of a case study. Finally, recommendations for future nursing research on this model are provided.

## 2. Background

In recent years, two significant developments in nursing research have influenced nursing research and practice. First, the focus on Patient Centred Care (PCC), sometimes referred to as person-centred care [14], has underpinned the development of the PPM-NI. PCC, which is partnering “with” patients, rather than providing services “to” them, and is advocated as a way to foster therapeutic relationships between patients, care providers and family [14]. Importantly, PCC is underpinned by values of respect for the individual, and the promotion of the patient’s self-determination, understanding and mutual respect [14]. PCC therefore encourages patient autonomy and input into decision making, individualising patient care and involving patients in a dialogue about their care [15,16]. This broad orientation towards patients can guide nurses in their practice, but such an orientation is often theoretical. While staff may state that they use a PCC approach, without a method of implementation it is possible they may use the language without the clinical care being underpinned by the values of the PCC approach. The PPM-NI is directed at filling this gap, and can be described as a situation-specific model to promote PCC.

A second significant development is nursing research—the focus on the patients’ strengths and capabilities—has also underpinned the development of the PPM-NI. A Capabilities Approach (CA) to care provides a foundation that conceptualises quality of life as a target toward which caregiving efforts should strive [17,18]. The CA belongs to the theories of human flourishing. It ratifies the beliefs that illness and disability, for example are socially brokered, and can interfere with the person’s ability to make choices, to be valued and to participate as a full member of society [17]. This approach values the individual choice and their opportunity to participate as a full member of society. Feeling valued is central to providing opportunities for the individual to live life well no matter whether the individual has a disability. The CA considers the factors necessary for patients to experience optimal well-being by focusing on opportunities that will enable the patient to experience their highest possible functioning. Family or significant other participation in care and decision making also helps to maintain a patient-centred approach. It is imperative that a “one size fits all” approach is avoided.

In addition to these two healthcare trends, because the focus of the PPM-NI is on the development, testing and subsequent adoption of nursing interventions, the literature on complex healthcare interventions has influenced its development. Specifically, the increasing focus on the impact of nursing care on patient outcomes has seen the emergence of research into the interventions nurses undertake and the patient outcomes that may be sensitive to these interventions, sometimes termed nursing-sensitive outcomes or indicators [19,20]. It is now well recognised that nursing interventions frequently entail multiple factors, and thus fall within the realm of complex healthcare interventions. Complex healthcare interventions are interventions that contain several interacting components [11,12,21]. This complexity can extend to what is expected of those delivering the intervention, the intervention target (*i.e.*, individual, group, organisation) and the degree of flexibility or tailoring of the intervention that is permitted [11,13,21]. A distinguishing feature of complex healthcare interventions is that they are known to involve behaviours, either overseen by the person delivering the intervention and/or displayed by the recipients of it [21]. The Medical Research Council (UK) recommend three inter-related activities required to develop good quality complex interventions, including: (1) identifying existing evidence; (2) identifying and developing theory to underpin the intervention; and (3) modelling the intervention prior to full scale testing [13]. Our development of the PPM-NI is directed at this second activity, of developing theory to underpin nursing interventions.

## 3. The Partnering with Patients Model of Nursing Interventions (PPM-NI)

**Figure 1:**
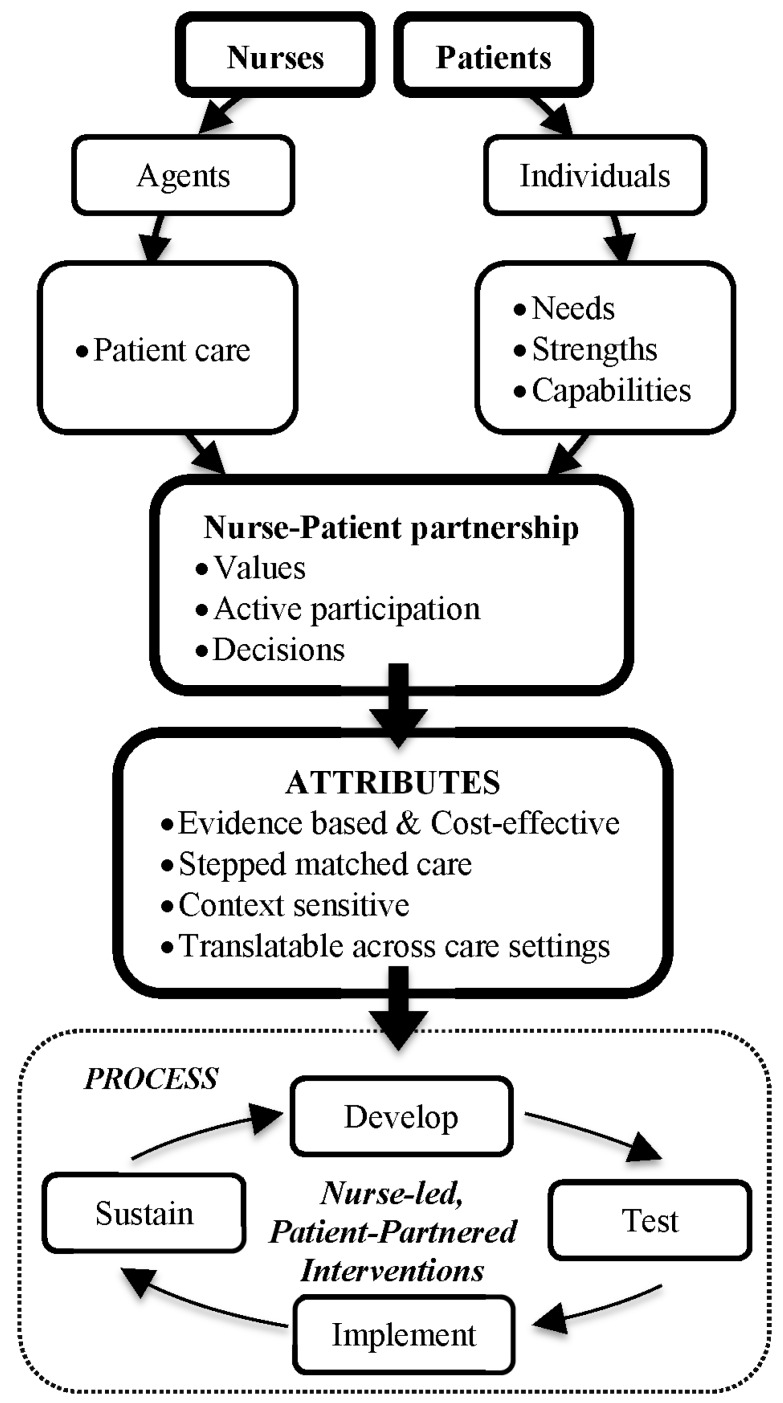
The partnering with patients model of nursing interventions.

Coalescence of our knowledge about PCC, the CA and complex nursing interventions has led to the development of the following assumptions underlying the PPM-NI, which is represented in Figure 1.

The assumptions of the PPM-NI are:

1. People are valued as individuals. As such, their individual strengths and capabilities need to be understood, as these will facilitate active participation in their care;
2. Nurses are ‘agents’ who have the capacity to make decisions and act on them to meet the needs of individual patient care (*i.e.*, to deliver an intervention); and
3. Nursing care centres on the needs of the person in the moment and how these needs can be met through the care partnership.

Based on these assumptions, the concepts (*i.e.*, characteristics) of nursing interventions grounded in the PPM-NI model are:

1. Evidence based;
2. Cost effective;
3. Patient centred, delivered by nurses in partnership with patients;
4. Stepped or tailored to the individuals’ needs, capabilities, goals and to the context, including family involvement;
5. Developed and tested in collaboration with end-users (*i.e.*, patients and nurses);
6. Applicable across settings; and
7. Multi-level, adapted to different levels of intervention targets (patient, ward/department, organisation).

Table 1 provides provisional descriptions of these concepts. Nurses may use these concepts and factors to help with their decision process when they are weighing up the benefits of interventions they may use. As a result, the propositions that arise from these concepts are that nursing interventions are more likely to be implemented and sustained in practice when they are based on the concepts (*i.e.*, characteristics) outlined above.

Although the PPM-NI requires further testing, it can readily be articulated into practice. The following case study demonstrates the potential of this model in practice and its potential to influence positive change. Furthermore, the case study demonstrates the importance of the factors outlined in Table 1 when considering whether to use a complex intervention.

## 4. Case Study: Joan

Background: Joan is 62 years old, has a diagnosis of early onset dementia and has been newly admitted to a long-term care facility, as her husband has a physical disability due to severe arthritis and is unable to manage her care. Joan spends her day wandering the facility, agitated and distressed, often crying and unable to be consoled. Staff and other residents are concerned by the distress that Joan exhibits but do not know how to help her. The manager asks a team from the university who have been exploring the use of companion animal robots if they can trial one of the companion robots with Joan. The research was approved by the University Ethics Board and forms part of a larger body of work being conducted by Moyle.

**Table 1:** Factors to consider when designing PPM-NI based interventions.

Concept	Factors
Evidence-based	What level of evidence is available to support the intervention? To what extent does expert consensus guide the intervention? Is there emerging empirical data to support the proposed approach?
Cost effective	Considerations of intervention costs and outcomes for patient/family to demonstrate “value for money” and increase sustainability. To what extent are efficiencies evident in the use of resources?
Active Partnership	How do both nurses and patients influence the intervention process? Is this process bi-directional? To what extent are patient and nurse values considered in the intervention approach? How is this expressed? Are values explicit or implied?
Stepped or Tailored care	Does the intervention respond to patient need in a systematic way? What algorithms and stepping rules are available to direct care in a patient centred approach? To what extend are family involved?
Developed and tested with end-users	Were end-users involved in the development and testing of the intervention? How did end users influence the intervention?
Translatable across settings	To what extent can the intervention be translated across settings that may vary by geography; care focus; and culture?
Multi-level	To what extent does the intervention respond to the context of both the patient and the nurse? Is there a clear pathway to develop, test, implement and sustain the intervention?

**Figure 2:**
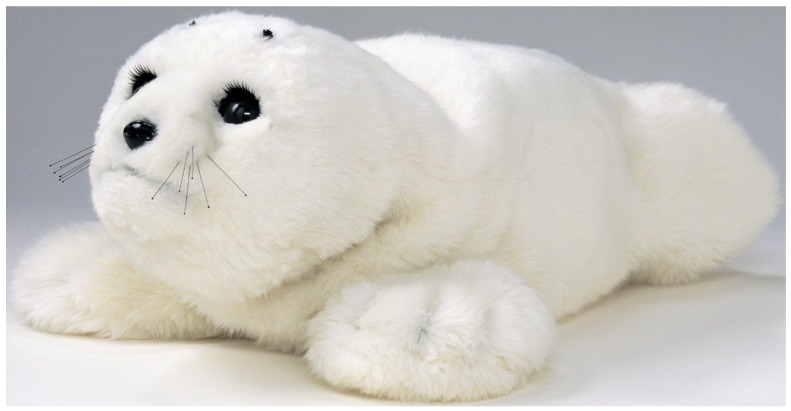
Paro.

Evidence based and cost effective: Although the use of companion robots is a relatively new area of study, empirical research has found that people with dementia may retain affective capability and can react positively to stimuli such as communication with robotic animals. Interaction with robotic animals has been shown to have a positive psychological effect on some people with dementia, improving mood, motivation, socialization, quality of life and reducing anxiety [22,23]. Moyle leads a research agenda exploring the effect of companion animal robots with people with dementia. The robot presented in this case study is Paro, which is an emotional robot in the form of a baby harp seal developed in Japan by Takanori Shibata [23] (see Figure 2). The Paro are expensive and one case study cannot indicate the cost effectiveness of this approach. However, the collective research aims to identify the cost effectiveness of the robot in care of people with dementia.

Paro is covered in tactile sensors that detect Paro’s position and temperature, vision and hearing which also react when the robot is being touched. Actuator motors are positioned in the eyelids, allowing the eyelids and eyes to react to all senses, and in the upper body, front paw and hind limbs, allowing Paro to move its flippers in reaction to being touched, stroked and spoken to. The developer programmed both pro-active and reactive behaviours, which enable Paro to interact with people in a very life like animal manner. The objective is for Paro to encourage interaction that contributes through stimulation to a person’s wellbeing, entertainment, and engagement.

Recent research into Paro compared the effect of the Paro to participation in an interactive reading group in people with moderate to severe dementia [22]. Participants were randomised to a Paro group intervention activity for 45 min, three times a week for five weeks or to an interactive reading group of the same length. The effect of the Paro intervention was assessed using the standardised difference in means [24]. The Paro intervention had a positive clinically meaningful influence on quality of life, emotions, pleasure and anxiety [22].

Patient-Centred, Delivered by Nurses in Partnership with Patients: The team talked with Joan, the care staff and Joan’s husband and found Joan has a love of animals. She recently lost her dog and her husband believes Joan’s distress has been worse since the death of the animal. It is decided to approach Joan and ascertain her interest and desire to interact with Paro. Naturally, if Joan appeared disinterested or had a negative response, the intervention would be discontinued and other interventions considered. This is important in the determination of the patient-centred goals and motivations, and consistent with the model.

Stepped or tailored to the individuals’ capabilities and to the context, including family involvement/ developed and tested in collaboration with end-users (*i.e.*, patients and nurses): Staff are trained by the team to deliver Paro as an intervention for Joan’s distress, to monitor the effect of the robot and to sensitively remove the robot. Staff are also taught to video the initial sessions so that the team and staff can discuss the intervention and identify any potential means to improve the intervention. As per the Ethics Board approval, consent to video Joan was obtained from the family.

When first given Paro, Joan looks at it inquisitively. The staff member works with Joan helping Joan to discover what Paro can do. Joan smiles, laughs and places Paro on her shoulder stroking the robot affectionately. The robot responds and nuzzles into Joan and she smiles and relaxes in the chair. After the first week, staff begin to recognise situations where Joan becomes distressed and they begin to present Paro before Joan is distressed. The robot is used in this case for comfort and as a non-pharmacological means to reduce Joan’s anxiety. The intervention is a success for Joan in relieving her distress and furthermore encouraged staff to be more aware of triggers of Joan’s anxiety, thus providing a truly patient-centred approach to Joan’s care. It can be used more or less often, and for shorter or longer durations of use, depending on Joan’s individual needs.

Applicable across settings/multi-level, adapted to different levels of care (patient, ward, organisation): Although the robot in this case was used in a long-term care setting, the intervention has applicability across acute and subacute settings where there are growing numbers of older people being admitted with cognitive impairment. At the group level, other residents also were noticed to be smiling and watching Joan interact with Paro, when she was in shared areas. Hence, the intervention also produced a positive effect for the social environment that was reinforcing for both other residents and staff.

## 5. Implications for Nursing Practice

The PPM-NI provides a potentially useful lens for clinical nurses to reflect on their practice and to confirm or re-negotiate their professional identity. Currently, nurses may be aware of the modern movements of PCC care or the CA, may even believe that their practice is consistent with these philosophies. However, because these are relatively new concepts that were not in the educational preparation for educational programs undertaken by many currently practicing nurses, there may not be widespread in-depth understanding of, or true integration into practice of these concepts. Practitioners often have difficulty with the application of existing professional theories to practice, as these can seem overly academic or esoteric to clinical nurses [25]. Because the PPM-NI model is based on nursing interventions as the distinct link between patients and nurses, it has perhaps a stronger likelihood of being interpretable by and attractive to practicing nurses.

## 6. Recommendations for Nursing Research

A model is a beginning step in theory construction. Models provide frameworks to organise phenomena and their relationships; an alternate way of viewing some subject matter. Research into the use of the model in several contexts allows its refinement and the development of situation-specific theories, and ultimately the development of testable hypotheses. We encourage the active discussion and debate of this model in the professional literature and we maintain that the use of case studies, as applied here, are highly useful in the development of usable and complete conceptual models and their component relationships. We are hopeful that nurse researchers across the venues of practice—acute, community and aged care, will all contribute to the further development of this model so that its relevance and utility to all will be maximised.

To operationally test the PPM-NI model, we suggest three main areas of investigation and data analysis that will be necessary to validate this model. These are:

1. Patients’ perceptions and experiences: Research is needed to understand whether the consumers of nursing interventions can recognise when a PPM-NI approach to their nursing care is experienced. This may be aided by associated work, for example, a comprehensive review of instruments to measure person-centred care noted that while there is no universally accepted definition of the term, and approaches to measure it (or its components) included patient preferences, patient experiences and patient outcomes [26]. Methods to better understand PCC include the use of surveys, interviews (either group or individual) and observation [26]. It may be that for both qualitative research and quantitative research, measuring PCC may help to inform future developments in the PPM-NI.
2. Nurses’ perceptions and experiences: Research is also needed to assess nurses’ perception of the applicability of the PPM-NI model to their practice, and to more deeply explore the various concepts in the model and their inter-woven relationships. It may be for example that certain aspects of the model require further refinement, or the relationships between concepts are more complex or multifactorial than we have described here. Integrated knowledge translation, whereby potential research knowledge users are engaged in the entire research process, may be a useful approach [27]. The use of process evaluations alongside the testing of new interventions based on the PPM-NI model may be useful to gain nurses’ perceptions and experiences. Process evaluations [28], also referred to as realist evaluations [29], help to better understand how, for whom and under what conditions our interventions work [28,29]. That is, involving end users in efforts to test and refine the PPM-NI may be warranted.
3. Outcomes of PPM-NI consistent care: Research is needed to test whether PPM-NI based nursing interventions are more effective than care that is not reflective of this model, measured both at the health outcome level, and also at a resource use/cost-effectiveness measure. We hypothesise that PPM-NI care will ultimately lead to lower health resource use of both nursing time and intervention costs because more appropriate and timely care will be delivered. In this area, in addition to using explanatory randomised controlled trial (RCT) design to test efficacy, pragmatic trials to test effectiveness may be particularly valuable [30,31]. Pragmatic trials are particularly well suited for testing nursing interventions in several ways. First, they focus on effectiveness in usual circumstances or practice. Second, interventions are applied in a flexible way, as they would be in clinical practice. Finally, research findings are generally directly relevant to patients, clinicians and decision makers. Sackett suggests that pragmatic trials answer the question “Does this treatment improve patient-important outcomes when applied by typical clinicians to typical patients?” [30].

## 7. Conclusions

A scientific discipline is distinguished by its unique body of knowledge, developed from both research and theory. We present here for the first time a new model for driving and understanding modern nursing practice, which is based on the concepts of PCC, the CA and the ability of nurses to join with patients in the delivery of complex interventions that are stepped in complexity and are appropriate to the moment in time. Once articulated, to be beneficial, models require further refinement through empirical testing, use and debate.
